# Roles of obesity in mediating the causal effect of attention-deficit/hyperactivity disorder on diabetes

**DOI:** 10.1017/S2045796023000173

**Published:** 2023-05-11

**Authors:** Ningning Liu, Jiang-Shan Tan, Lu Liu, Haimei Li, Yufeng Wang, Yanmin Yang, Qiujin Qian

**Affiliations:** 1 Peking University Sixth Hospital/Institute of Mental Health, Beijing, China; 2 NHC Key Laboratory of Mental Health (Peking University), National Clinical Research Center for Mental Disorders (Peking University Sixth Hospital), Beijing, China; 3 Emergency and Critical Care Center, State Key Laboratory of Cardiovascular Disease, Fuwai Hospital, National Center for Cardiovascular Diseases, Chinese Academy of Medical Sciences and Peking Union Medical College, Beijing, China

**Keywords:** ADHD, diabetes, Mendelian randomization, obesity

## Abstract

**Aims:**

Previous observational studies have reported potential associations among attention-deficit/hyperactivity disorder (ADHD), obesity, and diabetes (including type 1 and type 2 diabetes mellitus [T1DM/T2DM]). However, whether the association between ADHD and diabetes is mediated by obesity is unknown.

**Methods:**

With two-sample Mendelian randomization, we analysed the causal effect of ADHD on T1DM and T2DM and six obesity-related traits [including body mass index, waist circumference (WC), hip circumference, waist-to-hip ratio (WHR), body fat percentage and basal metabolic rate] and the causal effect of these obesity-related traits on T1DM/T2DM. Finally, with multivariable Mendelian randomization, we explored and quantified the possible mediation effects of obesity-related traits on the causal effect of ADHD on T1DM/T2DM.

**Results:**

Our results showed that ADHD increased the risk of T2DM by 14% [odds ratio (OR) = 1.140, 95% confidence interval (CI) = 1.005–1.293] but with no evidence of an effect on T1DM (OR = 0.916, 95% CI = 0.735–1.141, *P* = 0.433.). In addition, ADHD had a 6.1% increased causal effect on high WC (OR = 1.061, 95% CI = 1.024–1.099, *P* = 0.001) and an 8.2% increased causal effect on high WHR (OR = 1.082, 95% CI = 1.035–1.131, *P* = 0.001). In addition, a causal effect of genetically predicted high WC (OR = 1.870, 95% CI = 1.594–2.192, *P* < 0.001) on a higher risk of T2DM was found. In further analysis, WC mediated approximately 26.75% (95% CI = 24.20%–29.30%) of the causal association between ADHD and T2DM.

**Conclusions:**

WC mediates a substantial proportion of the causal effect of ADHD on the risk of T2DM, which indicated that the risk of T2DM induced by ADHD could be indirectly reduced by controlling WC as a main risk factor.

## Introduction

Attention-deficit/hyperactivity disorder (ADHD) is a highly heritable childhood behavioural disorder that can cause significant effects on individuals with ADHD and their families. Recently, an increasing number of studies have shown that individuals with ADHD have a higher risk of developing endocrine and metabolic disorders, and diabetes mellitus (DM) is one of the most common of these disorders (Akmatov *et al.*, 2021; Chen *et al.*, 2013, 2018a). However, over the last decade, multiple cross-sectional and longitudinal studies have indicated a significant association between ADHD and obesity, which is another most frequent endocrine and metabolic disorders (Cortese *et al.*, 2016; Cortese and Tessari, 2017; Nigg *et al.*, 2016). Notably, obesity is considered one of the strongest risk factors for the development of diabetes. Thus, there has been much speculation that ADHD indirectly affects the risk of diabetes mediated by obesity.

However, no studies could furnish any direct evidence for the question above. In addition, it is worth noting that even with many observational research, they could not establish a temporal relationship and causal inference, and other potential confounding factors might have also biased the results. For example, physicians may prescribe medications for ADHD that induce hyperglycaemia in rats and might contribute to the risk of DM (Arnerić *et al.*, 1984). Therefore, whether there is a causal relationship between ADHD and DM, and whether the relationship is directly or indirectly mediated through the increased prevalence of obesity are still unknown.

Understanding this relationship could be of great significance for clinical treatment. DM can cause deleterious effects in multiple organs and lead to a wide range of serious medical complications (Zheng *et al.*, 2018). If the relationship between ADHD and DM is mostly mediated indirectly by the increased prevalence of obesity as a main risk factor, no extra attention is required for the risk of DM for patients with ADHD. In contrast, more attention should be given to DM caused by ADHD if ADHD directly leads to DM. However, no direct evidence is available on this question, although much clinical and basic research has been performed.

Mendelian randomization (MR) is a newly emerged genetic epidemiological method that uses genetic variants as instruments to estimate the effect of an exposure on an outcome of interest. In addition, MR can be extended to estimate direct effects, indirect effects and proportions mediated. Unlike traditional observational mediation analysis approaches, MR estimates are robust to violations of the often untestable assumptions of the noninstrumental variable between an exposure, mediator or outcome, including unmeasured confounding and measurement error (Carter *et al.*, 2021).

Therefore, based on international genetic consortia, we investigated the effect of ADHD on DM by two-sample MR and the role of obesity in mediating the causal effect of ADHD on the risk of DM by multivariable MR (MVMR). Most of the previous research used body mass index (BMI) as an obesity marker. However, given that a recent study proposed that BMI alone cannot help adequately assess and manage all obesity-related health risks (Ross *et al.*, 2020), we chose multiple other obesity-related traits in the current study beyond BMI, including waist circumference (WC), hip circumference (HC), waist-to-hip ratio (WHR), body fat percentage (BFP) and basal metabolic rate (BMR). Considering the different aetiologies and physiopathology of type 1 DM (T1DM) and type 2 DM (T2DM), we studied them separately in the current study. To obtain the most robust conclusions, we performed adequate sensitivity analysis, including weighted median, MR‒Egger, heterogeneity, horizontal pleiotropy and MR pleiotropy residual sum and outlier (MR-PRESSO) methods. We hope this study could help us understand the mechanisms by which ADHD affects DM and provide a reference for determining public health policies.

## Methods

### Overall study design

**Figure 1.**
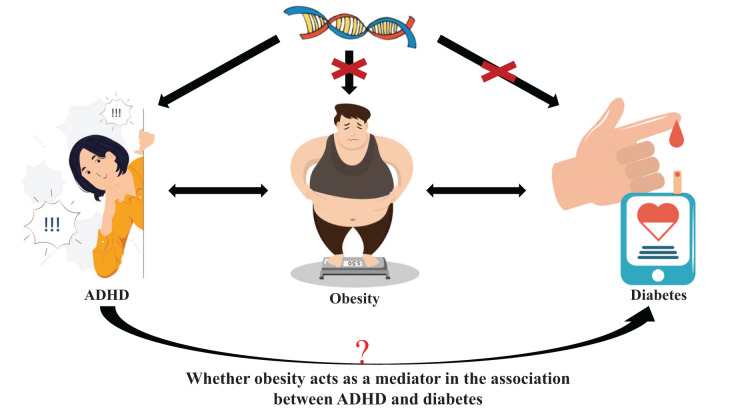
Schematic representation of an MR analysis. We selected SNPs associated with ADHD and the corresponding effect for these SNPs was estimated based on the risk of obesity or diabetes. Because of the randomization and independence of alleles at meiosis, MR is a powerfully predictive tool to assess causal relationships with no bias inherent to observational study designs. Besides, the present MR was used to estimate whether obesity acts as a mediator in the causal association between ADHD and diabetes.

The summary data of the present MR analysis are openly available from public databases, for which ethical approval has been obtained from the Ethics Committee in their respective studies. Therefore, ethical approval was not needed in this MR analysis. This study was conducted in three steps. The first step was to determine the causal effect of ADHD on T1DM/T2DM and six obesity-related traits. The second step was to determine the causal effect of these obesity-related traits on T1DM/T2DM. The third step was to explore and quantify the possible mediation effects of the six obesity-related traits on the causal effect of ADHD on T1DM/T2DM (Fig. 1).

### Data sources

If more than one previously published genome-wide association study (GWAS) dataset was available in the database, we chose the largest or the newest one with detailed publication information (including authors and year of publication) for further analysis. The characteristics of the included studies are shown in Table 1.

**Table 1.** Characteristics of selected genome-wide association studies

Trials	Cases (N)	Controls (N)	Sample size (N)	Year of publication	No. of SNPs
ADHD (Demontis *et al.*, 2019)	20,183	35,191	55,374	2017	8,047,420
Waist circumference^a^	–	–	462,166	2018	9,851,867
Hip circumference^a^	–	–	462,117	2018	9,851,867
Waist-to-hip ratio (Shungin *et al.*, 2015)	–	–	224,459	2015	2,562,516
Body mass index (Yengo *et al.*, 2018)	–	–	681,275	2018	2,336,260
Body fat percentage^a^	–	–	454,633	2018	9,851,867
Basal metabolic rate^a^	–	–	454,874	2018	9,851,867
Type 1 diabetes (Forgetta *et al.*, 2020)	9,266	15,574	24,840	2020	12,783,129
Type 2 diabetes (Bonàs-Guarch *et al.*, 2018)	12,931	57,196	70,127	2018	14,277,791

a Output from GWAS pipeline using Phesant-derived variables from UKBiobank.

ADHD, attention-deficit/hyperactivity disorder

#### Genetic instrumental variables for ADHD

The genetic variants for ADHD were obtained from the Psychiatric Genomics Consortium, which consists of 8,047,420 genetic variants of European ancestry and includes 20,183 cases and 35,191 controls (Demontis *et al.*, 2019). The related characteristics of the ADHD summary data are available at https://gwas.mrcieu.ac.uk/datasets/ieu-a-1183/.

#### Genetic instrumental variables for obesity-related traits

Genetic variants of WC, HC, WHR, BMI, BFP and BMR were extracted from a genetic analysis of 462,166, 462,117, 224,459, 681,275, 454,633 and 454,874 individuals of European ancestry, respectively. Detailed information is shown in Table 1.

#### Genetic instrumental variables for T1DM/T2DM

The summary statistics for T1DM were obtained from a GWAS with 12,783,129 genetic variants in 9,266 cases and 15,574 controls from European cohorts (Forgetta *et al.*, 2020), which is available at https://gwas.mrcieu.ac.uk/datasets/ebi-a-GCST010681/. T2DM was obtained from a GWAS with 14,277,791 genetic variants in 12,931 cases and 57,196 controls of European ancestry (Bonàs-Guarch *et al.*, 2018), which is available at https://gwas.mrcieu.ac.uk/datasets/ebi-a-GCST005413/.

### Statistical analysis

#### Step 1. Determine the causal effect of ADHD on obesity-related traits and T1DM/T2DM

The criteria for selecting SNPs for ADHD, obesity-related traits and T1DM/T2DM were as follows: (1) the SNPs were significantly associated with ADHD, obesity-related traits and T1DM/T2DM with genome-wide significance (*P* < 5 × 10^−8^) in each respective cohort study; (2) to avoid offsets caused by linkage disequilibrium (LD), SNPs had to be independent of each other by satisfying the LD of SNPs associated with ADHD, obesity-related traits or T1DM/T2DM with a threshold of *r*^2^ < 0.001 and window size = 10,000 kb. The LD levels were calculated based on the 1000 Genomes Project of European samples (Abecasis *et al.*, 2010).

The causal effects of ADHD on obesity-related traits and T1DM/T2DM were estimated using a two-sample MR method. In the MR analysis, traditional inverse variance weighting (IVW) was considered the most effective method (Burgess *et al.*, 2013). However, the IVW method is based on the assumption that all of the instrumental variables involved in the analysis are valid. Bias will be generated if not all SNPs satisfy this assumption. Therefore, MR‒Egger (Bowden *et al.*, 2015) and weighted median (Bowden *et al.*, 2016) were used as sensitivity analyses in the present MR analysis. The weighted median method requires the SNPs to satisfy the assumption that at least 50% of instrumental variables were valid. The involved SNPs are arranged based on their weight. Then, the median of the corresponding distribution function is defined as the result of the weighted median analysis. The valid effect estimate of MR‒Egger regression does not rely on any pleiotropic effects.

The pleiotropic effects can be estimated by the intercept of MR‒Egger. The MR‒Egger intercept test showed no significant difference from zero with a *P* value ≥ 0.05, and therefore, there was no evidence for directional pleiotropic effects. In addition, MR-PRESSO was used to further detect directional pleiotropic effects by removing SNPs with pleiotropic outliers (*P* < 0.05) (Verbanck *et al.*, 2018). The consistency of the IVW (*P* < 0.05), MR‒Egger and weighted median methods (similar estimation with IVW) with no significant directional pleiotropic effects (*P* > 0.05) suggested a causal effect of ADHD on obesity-related traits and DM. The odds ratio (OR) and 95% confidence interval (CI) were used to display our results.

Our MR analysis was performed by the TwoSampleMR package (version 0.5.5, https://gwas.mrcieu.ac.uk) and MR-PRESSO (version 1.0) of R version 3.6.0 (2019-04-26). The data and codes for this study can be obtained from the corresponding author on reasonable request.

To account for multiple testing in our primary analyses of the six obesity-related traits, a Bonferroni-corrected threshold of *P* < 0.008 (*a* = 0.05/6 outcomes) was used. A *P* value of 0.008 ≤ *P* < 0.05 was regarded as a suggestive finding.

#### Step 2. Determine the causal effect of obesity-related traits on T1DM/T2DM

The estimates of the effects of obesity-related traits on T1DM/T2DM were obtained by regression-based MVMR (Bowden *et al.*, 2015). The results are reported using ORs and 95% CIs. The judgement of causal association was the same as in Step 1.

#### Step 3. Mediating effects of obesity-related traits on the causal association between ADHD and T1DM/T2DM

To obtain the mediation effects of obesity-related traits on the causal role of ADHD on T1DM/T2DM, an estimate of the effect of ADHD on the obesity-related traits was multiplied by an estimate of the effect of the obesity-related traits on T1DM/T2DM. Then, we divided the mediation effect by the total causal effect of ADHD on T1DM/T2DM to obtain the proportion mediated by obesity-related traits.

## Results

### Total effect of ADHD on DM

The characteristics of SNPs are shown in the Supplementary materials. All the IVs associated with ADHD or obesity-related traits are significant with reference to a level of genome-wide significance (*P* < 5 × 10^−8^).

**Figure 2.**
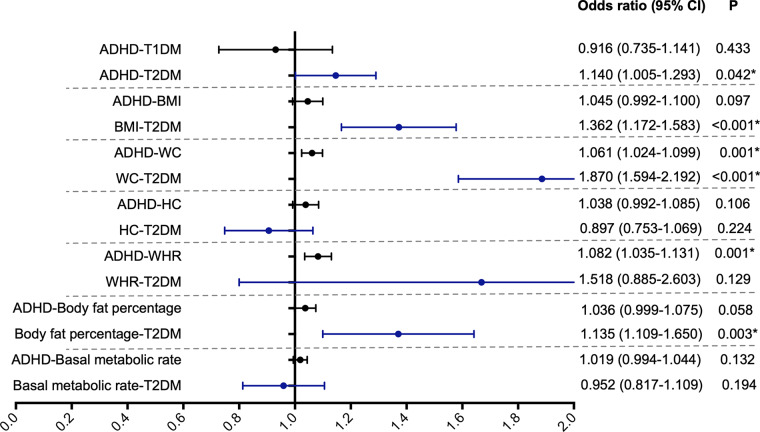
The potential causal association among obesity-related traits, diabetes and attention-deficit hyperactivity disorder.

Genetically predicted ADHD was causally associated with a higher risk of T2DM (OR = 1.140, 95% CI = 1.005–1.293, *P* = 0.042; Fig. 2) by the IVW method. However, we found no evidence of ADHD having a causal effect on T1DM (OR = 0.916, 95% CI = 0.735–1.141, *P* = 0.433).

Because no causal association was found between ADHD and T1DM, only the potentially mediating effects of different obesity measures on the causal association between ADHD and T2DM were further analysed.

### Effect of ADHD on obesity-related traits

In the present MR analysis, the IVW method showed that ADHD had a 6.1% increased causal effect on high WC (OR = 1.061, 95% CI = 1.024–1.099, *P* = 0.001) and an 8.2% increased causal effect on high WHR (OR = 1.082, 95% CI = 1.035–1.131, *P* = 0.001) (Fig. 2).

### Effects of obesity-related traits on T2DM

There was a causal effect of genetically predicted high BMI (OR = 1.362, 95% CI = 1.172–1.583, *P* < 0.001; Fig. 2), WC (OR = 1.870, 95% CI = 1.594–2.192, *P* < 0.001; Fig. 2) and BFP (OR = 1.135, 95% CI = 1.109–1.650, *P* = 0.003; Fig. 2) on the higher risk of T2D in the IVW method.

Because we found “ADHD→T2DM”, “ADHD→WC” and “WC→T2DM” in the previous analysis, we considered WC to mediate the relationship between ADHD and T2DM. Therefore, the mediating effect of WC on the association between ADHD and T2DM was analysed in the following sections.

### Mediation effects of WC on T2DM

In further analysis, we used WC for mediation analysis. After adjusting for ADHD, high WC was causally associated with an 81% increased risk of T2DM (OR = 1.811, 95% CI = 1.503–2.182, *P* < 0.001; Fig. 2). Therefore, the percentage of the causal effect of ADHD on T2DM mediated by WC was 26.75% (24.20%–29.30%).

### Sensitivity analyses

In the sensitivity analysis, the weighted median regression and MR‒Egger method showed directionally similar estimates but with low confidence. More details can be seen in Table 2. To further determine whether our horizontal pleiotropy was acceptable, the intercept of the MR‒Egger regression was used. As shown in Tables 3 and 4, the intercepts of the MR‒Egger regression analysis of all the causal effects did not show any horizontal pleiotropy (P > 0.05), suggesting no horizontal pleiotropy in the present MR analysis.

**Table 2.** The results of weighted median regression and MR–Egger methods in the present analysis

	Causal effect of ADHD on outcomes				Causal effect of obesity-related traits on T2DM			
	Weighted median		MR–Egger		Weighted median		MR–Egger	
Outcomes	OR (95% CI)	*P*	OR (95% CI)	*P*	Raw	*P*	Corrected	*P*
T1DM	0.969 (0.742−1.266)	0.819	0.623 (0.231−1.681)	0.377	–	–	–	–
T2DM	1.196 (1.004−1.426)	0.045	1.246 (0.709−2.191)	0.466	–	–	–	–
Body mass index	1.008 (0.979−1.037)	0.771	1.052 (0.758−1.460)	0.771	1.518 (1.281−1.798)	<0.001	1.433 (0.963−2.133)	0.077
Waist circumference	1.030 (1.005−1.056)	0.017	0.979 (0.837−1.146)	0.797	1.895 (1.527−2.353)	<0.001	1.213 (0.771−1.980)	0.405
Hip circumference	0.996 (0.969−1.023)	0.766	0.988 (0.802−1.215)	0.908	1.084 (0.912−1.289)	0.358	0.682 (0.422−1.102)	0.119
WHR	1.065 (1.010−1.123)	0.020	0.803 (0.656−0.983)	0.071	1.068 (0.672−1.698)	0.780	1.492 (0.151−14.760)	0.735
Body fat percentage	1.011 (0.991−1.032)	0.286	0.983 (0.831−1.162)	0.843	1.593 (1.271−1.996)	<0.001	0.779 (0.411−1.474)	0.443
Basal metabolic rate	0.944 (0.975−1.014)	0.557	0.957 (0.860−1.064)	0.439	1.043 (0.853−1.276)	0.682	0.759 (0.521−1.104)	0.150

CI, confidence interval; OR, odds ratio; WHR, waist-to-hip ratio; T1DM, type 1 diabetes; T2DM, type 2 diabetes; ADHD, attention-deficit/hyperactivity disorder.

**Table 3.** The results of heterogeneity, horizontal pleiotropy test and MR-PRESSO methods of attention-deficit hyperactivity disorder

	Heterogeneity test		Horizontal pleiotropy^a^		MR-PRESSO			
Outcome	*Q*	*P*	OR (95% CI)	*P*	Raw	*P*	Corrected	*P*
Body mass index	99.853	<0.001	0.999 (0.971−1.028)	0.968	1.045 (0.992−1.100)	0.135	1.033 (0.985−1.082)	0.315
Waist circumference	55.252	<0.001	1.007 (0.993−1.022)	0.334	1.061 (1.024−1.099)	0.010	1.046 (1.019−1.073)	0.009
Hip circumference	70.559	<0.001	1.005 (0.986−1.024)	0.644	1.038 (0.992−1.085)	0.140	1.004 (0.979−1.030)	0.765
WHR	12.339	0.137	1.027 (1.009−1.046)	0.021	1.082 (1.035−1.131)	0.008	–	–
Body fat percentage	78.702	<0.001	1.005 (0.990−1.020)	0.544	1.036 (0.999−1.075)	0.090	1.025 (1.000−1.050)	0.089
Basal metabolic rate	49.466	<0.001	1.006 (0.996−1.016)	0.267	1.019 (0.994−1.044)	0.167	1.017 (0.994−1.041)	0.198

CI, confidence interval; OR, odds ratio; MR-PRESSO, Mendelian randomization pleiotropy residual sum and outlier; WHR, waist-to-hip ratio.

a The MR–Egger intercept quantifies the effect of directional pleiotropy. *P* < 0.05 provides evidence that the exposure-associated single-nucleotide polymorphisms may influence the outcome through other pathways than through exposure.

**Table 4.** The results of heterogeneity, horizontal pleiotropy test and MR-PRESSO methods on T2DM

	Heterogeneity test		Horizontal pleiotropy^a^		MR-PRESSO			
Outcome	*Q*	*P*	OR (95% CI)	*P*	Raw	*P*	Corrected	*P*
ADHD	5.356	0.802	0.992 (0.943−1.044)	0.759	1.140 (1.034−1.257)	0.027	–	–
Body mass index	1209.666	<0.001	0.999 (0.993−1.006)	0.788	1.362 (1.172−1.583)	<0.001	1.459 (1.307−1.629)	<0.001
Waist circumference	648.565	<0.001	1.007 (1.000−1.014)	0.050	1.870 (1.594−2.192)	<0.001	1.991 (1.751−2.265)	<0.001
Hip circumference	1332.761	<0.001	1.005 (0.997−1.014)	0.231	0.897 (0.753−1.069)	0.224	1.066 (0.942−1.206)	0.309
WHR	141.491	<0.001	1.000 (0.944−1.060)	0.988	1.518 (0.855−2.603)	0.140	1.517 (1.000−2.297)	0.061
Body fat percentage	827.815	<0.001	1.008 (0.999−1.017)	0.075	1.353 (1.109−1.650)	0.003	1.715 (1.451−2.026)	<0.001
Basal metabolic rate	906.138	<0.001	1.003 (0.998−1.009)	0.194	0.952 (0.817−1.109)	0.529	0.962 (0.841−1.100)	0.570

ADHD, attention-deficit hyperactivity disorder; CI, confidence interval; OR, odds ratio; MR-PRESSO, Mendelian randomization pleiotropy residual sum and outlier; T2DM, type 2 diabetes mellitus; WHR, waist-to-hip ratio.

a The MR–Egger intercept quantifies the effect of directional pleiotropy. *P* < 0.05 provides evidence that the exposure-associated single-nucleotide polymorphisms may influence the outcome through other pathways than through exposure.

The heterogeneity test showed potential heterogeneity in most of our analyses. Therefore, MR-PRESSO global tests were used to correct potential heterogeneity. As shown in Tables 3 and 4, similar estimates were observed in the outlier-corrected results.

## Discussion

By using MVMR methods with large sample sizes, our genetic analyses found a causal effect of ADHD on the risk of T2DM, and further analysis revealed that 26.75% of the causal association between ADHD and T2DM is explained by WC. That is, WC mediated a substantial proportion of the excess risk of T2DM among patients with ADHD.

DM is a major worldwide cause of death and disability, and one person dies of DM every 5 seconds (https://diabetesatlas.org). Data from the International Diabetes Federation reveal that 537 million adults are living with DM globally, and the number is projected to increase to 783 million by 2045 (Cho *et al.*, 2018). Recent epidemiological studies suggest that individuals with ADHD suffer from DM more frequently than those without ADHD (Akmatov *et al.*, 2021; Xu *et al.*, 2021). Notably, most previous studies reported an increase in the rate of T2DM in participants with ADHD compared with controls but did not find increases in T1DM (Chen *et al.*, 2013, 2018a, 2018b). Indeed, it is known that the pathogenesis of T2DM is different from that of T1DM. T2DM is thought to be mainly caused by peripheral insulin resistance and relative insulin deficiency, whereas T1DM is primarily caused by autoimmune-mediated destruction of pancreatic β cells and insulin deficiency (Stumvoll *et al.*, 2005). A meta-analysis also indicated that patients with T2DM have medium to severe cognitive impairment, including memory and attention. However, patients with T1DM only have mild to moderate cognitive impairment (Samoilova *et al.*, 2020). These might explain why we only found an effect of ADHD on T2DM, rather than T1DM, in the current study. However, previous studies were based on observational studies. Other biases, such as unmeasured confounding, cannot be addressed by that method, which cannot reliably prove causality. In this study, we overcame these deficiencies and established a causal relationship between ADHD and T2DM by leveraging the power of GWASs.

Although many scholars have suggested that ADHD is associated with obesity through observational studies (Cortese and Morcillo, 2010; Cortese *et al.*, 2016), no consensus has been reached on the causal relationship between them. To solve this problem, later studies explored the causal relationships between ADHD and obesity with MR. Thais *et al*. found that higher BMI increases the risk of developing ADHD (Martins-Silva *et al.*, 2019). Beate *et al*. found that ADHD has a causal effect on childhood obesity (Leppert *et al.*, 2021). Later, to provide more comprehensive results, Ville *et al*. explored ADHD liability and six obesity-related traits and found causality between ADHD and BMI, WC, WHR and BMI-adjusted WHR (Karhunen *et al.*, 2021). Herein, we updated the dataset, which included more people, and confirmed the causal relationship between ADHD and the risk of WC. More importantly, we found that WC mediated the relationship between ADHD and T2DM.

In addition, some studies have revealed the possible mediating roles of obesity between ADHD and DM. Seymour *et al*. found some common neural correlates across ADHD and obesity, e.g., functional abnormalities within circuits subserving reward processing and executive functioning (Seymour *et al.*, 2015). Other studies have linked both obesity and ADHD to the dopamine system and suggested that dopaminergic changes in the prefrontal cortex may also increase the risk for obesity (Campbell and Eisenberg, 2007; Seymour *et al.*, 2015). Therefore, they believed that ADHD would lead to obesity. In addition, scholars have suggested that obesity is a major driver of pre-DM and DM (Boles *et al.*, 2017; Lüscher, 2020). Similarly, in another MR study, childhood and adulthood BMI, BFP and visceral fat mass were found to be associated with an increased risk of DM (Yuan and Larsson, 2020). Interestingly, previous studies found that patients with DM display abnormal brain structures, including frontal and parietal lobes (Liu *et al.*, 2020; Novak *et al.*, 2006; Zhou *et al.*, 2021), which have been considered to be the predominant brain areas underlying the pathophysiological mechanism of ADHD and obesity. These studies indirectly revealed the possible mediating roles of obesity between ADHD and DM. Although these studies proposed the assumption that ADHD has an underlying impact on DM through the indirect mediator of obesity, related observational mediation studies are rare.

Using a population-based dataset, Huiju *et al*. found that subjects with ADHD had a higher proportion of prior T2DM diagnoses than controls after adjusting for other potential confounding factors, including obesity (Chen *et al.*, 2013). Similarly, Guifeng *et al*. found that the association of ADHD with DM was still significant after adjusting for BMI (Xu *et al.*, 2021). Both studies rejected the hypothesis of an indirect effect between ADHD and DM. However, neither study took WC into account. In addition, even with such traditional mediation analysis, these studies are subject to the bias of measurement error. In contrast, MR studies could assess the lifelong exposure of alleles, which avoids reverse causality and reduces residual confounding factors, as the genetic variants were determined at conception before the onset of diseases. Therefore, the MR approach might offer favourable opportunities for understanding the mediational relationship.

In the current study, based on the evidence above and the strong hypothesis suggested earlier, we analysed the mediation effect with MR and found that the percentage of the effect of ADHD on the risk of DM mediated by WC was up to 26.75% (24.20%–29.30%). WC is one of the indices reflecting obesity and seems to be a less important indicator than BMI. However, a growing number of studies are beginning to recognize its importance. One study included 11,666 respondents and found a linear association between the number of inattentive and hyperactive/impulsive symptoms in adolescence and WC (Fuemmeler *et al.*, 2011). A recent study proposed that WC is associated with health outcomes in both categorical analyses and continuous analyses (Ross *et al.*, 2020) and might have a stronger ability to predict health outcomes than BMI. Another study found that impulsivity, which is one of the core symptoms of ADHD, is associated with WC in adolescents with bipolar disorder (Naiberg *et al.*, 2016). Fortunately, studies have found that clinically relevant reductions in WC can be achieved by routine, moderate-intensity exercise and/or dietary interventions (Ross *et al.*, 2020). That is, our study suggested that the risk of T2DM in individuals with ADHD can be greatly reduced with the control of WC, thus preventing diabetic complications and improving people’s quality of life.

## Limitations

As far as we know, this study is the first two-sample MR study to identify the mediation effects of obesity on the causal effect of ADHD on DM. To minimize bias from horizontal pleiotropy, we applied different MR sensitivity analyses, which produced consistent results with those from the main MR analyses. However, this paper still has some potential weaknesses. First, the data were mainly constrained to European populations, which might potentially limit the generalizability of our results to other populations and ethnicities. In addition, the genetic variants associated with ADHD were from a GWAS where ADHD was considered a binary trait, representing the average causal effect in “compliers” in MR analysis (Burgess and Labrecque, 2018). Although it does not indicate that testing for the causal null hypothesis is invalid (VanderWeele *et al.*, 2014), MR analysis conceptualized in terms of an underlying continuous variable is needed in the future. Finally, although our results found that WC mediated up to 26.75% of the relationship between ADHD and T2DM, there are other unknown additional mediating factors underlying the observed correlations between them that we cannot explain in this study. More research is needed to search for other mediating factors and further explore the underlying mechanism.

## Conclusion

By using distinct analytical methods, including genetic approaches that can draw a causal inference, our results confirmed for the first time that ADHD was associated with significantly higher odds of T2DM than T1DM. WC, which is one of the indices reflecting obesity that is often overlooked by policy-makers and researchers, mediates a large proportion of the association. Our study indicated that T2DM should be carefully considered in patients with ADHD, and controlling WC could help to reduce the risk of T2DM induced by ADHD.

## Data Availability

The datasets used and/or analysed during the current study are available from the corresponding author on reasonable request.

## Supplementary material

For supplementary material accompanying this paper visit http://doi.org/10.1017/S2045796023000173. click here to view supplementary material
